# Sex Differences in Behavioral Circadian Rhythms in Laboratory Rodents

**DOI:** 10.3389/fendo.2014.00234

**Published:** 2015-01-09

**Authors:** Jessica A. Krizo, Eric M. Mintz

**Affiliations:** ^1^Department of Biological Sciences, Kent State University, Kent, OH, USA; ^2^School of Biomedical Sciences, Kent State University, Kent, OH, USA

**Keywords:** estrogens, testosterone, locomotor activity, ovariectomy, castration

## Abstract

There is a strong bias in basic research on circadian rhythms toward the use of only male animals in studies. Furthermore, of the studies that use female subjects, many use only females and do not compare results between males and females. This review focuses on behavioral aspects of circadian rhythms that differ between the sexes. Differences exist in the timing of daily onset of activity, responses to both photic and non-photic stimuli, and in changes across the lifespan. These differences may reflect biologically important traits that are ecologically relevant and impact on a variety of responses to behavioral and physiological challenges. Overall, more work needs to be done to investigate differences between males and females as well as differences that are the result of hormonal changes across the lifespan.

## Introduction

There has been a longstanding bias against the use of females in basic research involving common animal models, arising out of the belief that females show higher variability in results due to the influence of hormonal cycles (1). However, a recent meta-analysis of publications involving the use of mice across a variety of biomedical research areas concluded that this assumption was without merit (2). The failure to include both females and males, therefore, can result in researchers missing important information on sex differences in biology without any resulting gains from limiting themselves to the use of a single sex. The study of biological rhythms is no exception to this critique, as a large majority of recent work in this area has failed to include females (3). In this review, we look at sex differences in basic parameters of circadian rhythms and hypothesize about their underlying mechanisms and biological relevance.

## Circadian Period

The period of the circadian clock represents the time it takes to complete one cycle under constant environmental conditions, and is usually close to but not exactly 24 h. Sex differences in period are highly species-specific, but even when present the differences are generally modest. Free-running period in rats and golden hamsters is longer in males than females (4, 5); however, the differences in period are very small, whereas in *Octodon degus* period is longer in females by approximately half an hour (6, 7). In mice with a C57BL/6J background, there does not appear to be a sex difference in free-running period (3).

Despite the limited nature of the sex differences, gonadal hormones have a significant impact on circadian period. Ovariectomy lengthens circadian period in rats and hamsters, and period is then shortened by replacement of estradiol (8, 9). However, no change in period is apparent in mice after ovariectomy (3), though estradiol, an estrogen receptor α (ERα) agonist, or an estrogen receptor β (ERβ) agonist shorten period in ovariectomized mice (10). In contrast, reports on the effect of castration on period are more varied. While one study showed no effect in mice (11), others indicate that it lengthens period in this species (12–14). It appears that this effect may be dependent on the presence of constant dim red light (as opposed to true constant darkness) (14). Castration does not result in a change in period in hamsters (15) or adult degus (16).

Given the modest, if any, sex differences in circadian period, it is appropriate to ask whether the differences that are seen are biologically relevant. There is natural variation in circadian period across species and individuals, but circadian period must be under stabilizing selection to keep it close to 24 h. Period length does influence the amplitude of responses of the circadian clock to stimuli (17), so average period for a species may reflect an optimization for responses to external stimuli rather than an optimization for period length. There does not seem to be an overarching theory that explains interspecies variation in period. It is likely that sex differences in period reflect the influence of gonadal steroids, either through direct action on the central circadian clock in the suprachiasmatic nucleus (SCN) or via actions that modify behavioral feedback on the SCN. Organizational effects of gonadal hormones may also play an important role, but in this review we are focusing on potential activational effects. That said, it is remarkable that sex differences are so small, given the considerable influence of gonadal steroids on circadian period.

## Onset of Activity

The timing of activity onset represents the most obvious difference in circadian rhythms between the sexes. Variability in activity onset is considerably greater in females than in males, and this variability is closely tied to the phase of the estrous cycle in mice (3), hamsters (8, 18, 19), and rats (20, 21). Variability in the onset of activity in females appears to be largely mediated by ERβ, at least in mice (10, 22). Activity onset is most advanced before ovulation, corresponding to elevated estradiol levels, and then delayed afterward. The functional significance, if any, of this variation is unknown. These effects could be caused by direct effects of estrogens on the phase of the underlying circadian clock and/or by changes in effector systems that cause the threshold for onset of locomotor activity to occur slightly earlier or later depending on the hormonal environment. The idea that the underlying clock in the SCN shifts a little on each day of the estrous cycle under the direct influence of estradiol is supported by the fact that period is shortened by replacement of estradiol in ovariectomized animals (8, 9, 23). However, it is also possible that clock output is unchanged, but downstream brain regions responsible for generating the motivation for locomotor activity are slightly more or less sensitive depending on the level of estrogens present. For example, estrogens upregulate dopamine receptor 1 in the striatum (24, 25), which could result in increased motivation for wheel-running activity, resulting in a slightly earlier onset of activity.

## Photic Responses

There are a number of potential mechanisms by which biological sex, via gonadal steroids, can influence the photic sensitivity of the circadian clock. However, it is not known if the effects that have been found thus far are biologically important, and these effects may vary dramatically by species. In *Octodon degus*, females adjust to a 6-h advance of the light–dark cycle significantly faster than males (26). In mice, females have larger phase shifts to light (3), while gonadectomized male mice have larger phase shifts than gonadally intact male mice (27). The lengthening of period that occurs when animals are housed in increasing intensities of constant light is also potentiated in gonadectomized animals (14). Female mice lacking estrogen receptor alpha show increased phase shifting responses to light (28). These data are consistent with the idea that both estradiol and testosterone act to reduce the phase shifting effects of light. The functional significance of this is unknown.

## Non-Photic Responses

There has been little work done investigating sex differences in non-photic influences on entrainment. A couple of studies have been done on the influence of the estrous cycle on circadian responses in Syrian hamsters, but not with direct comparisons to male animals. Females show an estrous-cycle dependent modulation of activity level in response to a non-photic stimulus such as a cage change or novel wheel exposure, but this difference in behavioral activation results in only modest variability in the size of non-photic phase shifts (29). However, they did note that large shifts during proestrus caused a 1-day delay in the estrous cycle. A similar delay was observed in response to phenobarbital treatment on proestrus, suggesting that large phase shifts caused the circadian clock to “miss” generating the daily signal needed for the GnRH surge (30). However, in order to demonstrate a true sex difference in non-photic responses, it will be necessary to conduct experiments with direct comparisons between males and females, and it is important that this be done in additional species to see if there are common responses. In degus, there are sex differences in the effect of odor on circadian reentrainment rates to shifts in the light/dark cycle, and these effects are influenced by estrogen, progesterone, and testosterone (31, 32).

## Food Entrainment

There has been very little research on sex differences in food entrainment. When rodents are placed on a restricted feeding schedule, such that food is only available for a limited period of time each day during an animal’s normal sleep period, they show a behavioral response known as food anticipatory activity (FAA). This FAA generally takes the form of increased behavioral activation for a period of about 3 h prior to food availability. FAA is particularly notable when animals are provided with a running wheel, as wheel-running during FAA can be more intense than normal nocturnal running. This activity is thought to be stimulated by the action of a circadian clock, as food availability that is timed in non-circadian intervals (e.g., 18 h) does not result in FAA (33). In addition, FAA persists for several cycles under conditions of total food deprivation after entrainment to timed restricted feeding, and also does so in the absence of the SCN (34, 35). Little work has been published concerning female responses to timed restricted feeding. Rats will entrain their activity rhythms to restricted feeding if in constant dark, but there is no evidence for or against a sex difference in the ability to entrain to restricted feeding. A few studies have investigated the role of the reward system on entrainment to feeding using palatable foods. To date sex differences in FAA have only been identified in mice. When receiving a high fat food as a snack, male mice exhibit anticipatory activity and females do not (36). However, females show activity at the time of previous food delivery on subsequent days, suggesting that the females are still timing the arrival of the food but are not showing the anticipatory activity. There is some evidence to suggest that female motivation for sugary/fat foods is modulated by the estrous cycle (37). This could impact the response of female mice to a palatable food cue during *ad libitum* conditions. The fact that circadian clock-driven anticipatory activity can occur under both normocaloric and hypocaloric conditions suggests that there are multiple drivers of FAA, a motivational circuit and a homeostatic circuit (38).

## Pubertal Effects on Rhythms

Puberty represents a period of substantial changes in physiology and endocrine profiles. Given that gonadal steroids have an impact on adult circadian behavior it is reasonable to hypothesize that the circadian system would be responsive to this dynamic endocrine environment. Pubertal changes in circadian phase have been noted in mice (39, 40) and rats (41, 42). However, these studies were limited to male or female subjects and therefore do not address the issues of sex differences. The role of gonadal steroids during the pubertal period on circadian development has been investigated in the rat and the degus (43–47). During the pubertal period, rats and degus (both male and female) have a bimodal distribution of locomotor activity during their active phase. By adulthood, activity in intact male rats and degus changes to a unimodal activity pattern. Pre-pubertal GDX in rats leads to a less extreme bimodal distribution that is maintained into adulthood, suggesting that gonadal steroids are responsible for the consolidation of activity to the beginning of the active phase. In males, GDX results in a loss or reduction in pubertal-related changes in circadian parameters, whereas GDX in females results in a more variable response (45). In degus, pre-pubertal GDX of both males and females stabilizes circadian phase and the bimodal distribution of activity persists into adulthood as seen in intact female degus (46). These studies taken together provide evidence for the developmental role of gonadal steroids during puberty in setting circadian behavioral rhythm parameters.

## Site of Action

A direct action of gonadal steroids on the SCN would be most likely be mediated by one or more of the steroid hormone receptors: ERα, ERβ, androgen receptor (AR), progesterone receptor (PR), or G protein-coupled estrogen receptor 1 (GPER1). ERα, ERβ, and AR are all expressed in the SCN (13, 48–51), with sexual dimorphisms present in ERβ, and AR (51). For a full review of the neuroanatomical aspects of sexual dimorphism in the circadian system, see (52). In addition, the SCN receives input from other estrogen receptor-positive regions of the brain (53), providing another potential mechanism for steroid-modulation of SCN function. Finally, it is possible that signals from some peripheral organs may be sexually dimorphic, and when activated they may alter rhythmic function in a sex-specific manner.

## Conclusion

There is a clear need for further research to understand how biological sex and gonadal hormones can regulate behavioral rhythmicity. Sex differences in basic behavioral activity rhythms are modest in scope; however, this may not be the case if the system is challenged. For example, there are substantial sex differences in the brain’s reward system (54) that could interact with circadian clocks in such a way that result in differential responses of the circadian clock to addictive drugs.

In general, the data reviewed in this article suggests that most initial research studies on the circadian system should be carried out using both male and female animals. If no sex differences in the results are observed, researchers can then decide whether their approach will work best using a single or mixed sex approach. For example, in experiments where precision of the onset of activity is critical, it may be appropriate to conduct studies in males, though modern mathematical techniques for ascertaining rhythm phase make this less of an issue than when activity rhythms were assessed by visual inspection of actograms. Failure to make use of both males and females in studies may result in important physiological and behavioral phenomenon remaining undiscovered.

Finally, most studies that look at female steroid hormone effects on circadian rhythms make use of experimental methods involving gonadectomy and hormone replacement. While such studies yield valuable information about the mechanisms of hormone influences on rhythms, they do not represent the normal physiological variation that occurs across a normal estrous cycle. It is understandable that this has occurred, given the increase in animal numbers needed and workload involved in measuring estrous cycle phase, however, such studies will become increasingly important as we learn more about the potential influence of gonadal hormones on behavioral circadian outputs.

## Conflict of Interest Statement

The authors declare that the research was conducted in the absence of any commercial or financial relationships that could be construed as a potential conflict of interest.
